# (Mesenchymal) Stem Cell-Based Therapy in Cisplatin-Induced Acute Kidney Injury Animal Model: Risk of Immunogenicity and Tumorigenicity

**DOI:** 10.1155/2017/7304643

**Published:** 2017-12-12

**Authors:** Ž. Večerić-Haler, A. Cerar, M. Perše

**Affiliations:** ^1^Department of Nephrology, University Medical Centre Ljubljana, SI-1000 Ljubljana, Slovenia; ^2^Institute of Pathology, Medical Experimental Centre, Faculty of Medicine, University of Ljubljana, Zaloška 4, SI-1105 Ljubljana, Slovenia

## Abstract

Pathogenesis of AKI is complex and involves both local events in the kidney as well as systemic effects in the body that are interconnected and interdependent. Despite intensive investigations there is still no pharmacological agent that could provide complete protection against cisplatin nephrotoxicity. In the last decade mesenchymal stem cells (MSCs) have been proposed as a potentially useful therapeutic strategy in various diseases, including acute kidney injury. Although MSCs have potent immunosuppressive properties, animal studies also suggest that transplanted MSCs may elicit immune response. Interestingly, tumorigenicity of transplanted MSCs in animal studies has been rarely studied. Since the risk of tumorigenicity of particular therapy as well as the immune response to solid or cell grafts is a major issue in clinical trials, the aim of the present paper is to critically summarize the results of MSC transplantation on animal models of AKI, particularly cisplatin-induced animal models, and to expose results and main concerns about immunogenicity and tumorigenicity of transplanted MSCs, two important issues that need to be addressed in future studies.

## 1. Introduction

Today, mesenchymal stem cell (MSC) therapy is recognized as a potentially useful innovative therapeutic strategy in various diseases [1]. Increasing number of experimental studies demonstrated beneficial effects of MSCs also in acute kidney injury (AKI) [2]. The pathophysiology of AKI is very complex and involves tubular and vascular cell damage and an intense inflammatory reaction. Current therapies of AKI mainly include supportive care, including renal replacement therapy. Despite these therapies, the five-year mortality rate for patients with AKI remains >50%. Hence, new therapeutic interventions and strategies for improving survival outcome for patients with AKI are needed. Stem cell-based therapy has gained great interest in AKI treatment over the years [2]. Recent studies have focused on the clinical efficacy of MSC transplantation [3]. However, in clinical trials, the immune response to allogeneic solid or cell grafts has always been a major issue [4, 5]. Although allogeneic MSCs have potent immunosuppressive properties, animal studies also suggest that they may elicit a weak allogeneic immune response [6].

Thus, the aim of the present paper is to critically summarize the results of MSC transplantation in animal models of AKI, particularly in cisplatin-induced animal models, and to expose important issues that need to be addressed in future studies. We have restricted our investigation on a cisplatin animal model, because it has specific characteristics that might have significant effect on short/long-term MSC studies.

To get insight into the reported side effects or risk factors of stem cell-based therapy in a cisplatin animal model, we conducted a PubMed search using keywords “cisplatin kidney and stem cells” and got 111 publications (July, 2017). Among them, 40 publications investigated the effects of stem cells on a cisplatin animal model and are shown in Tables 1 and 2.

Tables 1 and 2 show the source and type of stem cells used, immune state of the animals, duration of the studies, and potential short/long-term risk effects of stem cell transplantation, delivery route, and so on.

### 1.1. Important Factors to Consider before Conclusion Can Be Made

Although most of the studies using the cisplatin animal model reported that MSC transplantation ameliorates AKI, conclusions about the effectiveness and safety of MSCs must not be made before below stated factors are taken into consideration:

1. Characteristics of cisplatin animal models
2. Reliability of tracking the injected cells
3. MSCs and risk of immune rejection
4. MSCs and risk of tumorigenicity (duration of the study: most studies ended within a week, only few were performed to investigate potential side effects (8 weeks), but on very small number of animals (*n* = 3))

## 2. Characteristics of Cisplatin Animal Models

The cisplatin model has its own characteristics. It is important to take into consideration the dose used as well as its immunosuppressive and carcinogenic effects. When neprotoxic dose of cisplatin is used, kidney dysfunction develops in 2–5 days, reaching peak at 4–7 days and then progressively recovers (blood urea nitrogen/serum creatinine (BUN/Cr) reach the baseline levels). When lethal dose of cisplatin is used (Table 3), self-recovery is less likely. However, with the higher doses of cisplatin, survival time of animals markedly decreases [54]. Importantly, high-nephrotoxic doses of cisplatin in rodents lead to systemic side effects, such as body weight loss and mortality. Cisplatin usually causes diarrhea in all animals, a significant decrease in both the lymphocytes (65% decrease) and granulocytes (45% decrease) in the bone marrow, decrease in circulating peripheral white blood count (WBC) [49], massive necrotic changes in the kidney, injuries in the gastrointestinal tract, testis, bone marrow [44], and the lymph tissue [55]. Cisplatin is also carcinogenic and can cause lung tumors in rodents [56].

## 3. Reliability of Tracking the Injected Cells

Most of the studies used labeling to check or confirm the presence of injected cells in kidneys and/or other organs (see Tables 1 and 2). It was reported that labeled cells (PKH26, GFP, and DIO) were mostly detected in the lungs, much less in the liver and in the kidneys [8, 10, 14, 18, 20, 22, 23, 25]. Cheng et al. studied biodistribution and found that one hour after iv injection of syngeneic MSCs most of the radiolabeled (or GFP labeled) cells were trapped in the lungs (62%), followed by liver (12.5%), spleen (11.4%), and kidneys (5.4%), but 7 days after injection no signs of MSCs in any organ was found [36]. Studies using GFP labeling reported disappearance of GFP^+^ cells in the kidney 4 days after injection [22, 31], while studies that used PKH26 [12, 33] or CM-Dil [13] labeling reported presence of positive cells in the kidney until the end of their study, that is, 2–4 wks. Nevertheless, the fact that injected MSCs are mostly trapped in the lungs and cleared without any engraftment in kidney raise questions regarding their pathophysiologic mechanisms, as well as possibility of their potential rejection by the host's immune system.

## 4. MSCs and Risk of Immune Rejection

### 4.1. Can Xenogenic or Allogeneic MSCs Survive in Immunocompetent Environment?

Human MSCs express specific membrane antigens (CD73, CD90, and CD105) and intermediate levels of major histocompatibility complex (MHC) class I molecules, while, in a naive state, they do not express MHC class II and the costimulatory molecules CD80 (B7-1), CD86 (B7-2), CD40, or CD40L [57]. They should therefore be recognized by alloreactive T cells, but numerous *in vitro* studies have shown that undifferentiated and differentiated human MSCs escape recognition by alloreactive T cells, escape lysis by cytotoxic T cells and natural killer (NK) cells, and inhibit mixed lymphocyte cultures (MLC), [57–59], suggesting that MSCs may thereby circumvent rejection and can thus be transplantable between MCH-incompatible individuals without the need for host immunosuppression. Furthermore, the observation that MSCs are immunoprivileged and display immunosuppressive characteristics [60] suggest their therapeutic value in allogeneic transplantation to prevent graft rejection and to prevent/treat graft versus host disease.

Numerous experimental studies have reported that transplantation of allogeneic or even xenogeneic MSCs into immunocompetent animals without the use of immunosuppressants resulted in an improvement (reviewed in Lin et al. [61]) of a wide range of diseases, including cisplatin-induced AKI, suggesting that hMSCs are immunotolerant. However, although MSCs seem to be transplantable across allogeneic or even xenogenic barriers, some animal studies have clearly shown that the cellular and humoral responses against the xenogenic MSCs in an immunocompetent recipient can develop (some example are shown in Table 4). Results also show that allogeneic MSCs are not intrinsically immunoprivileged but under appropriate conditions induce T cell response resulting in rejection of an allogeneic stem cell graft [65].

### 4.2. MSCs Mechanism and Risk of Immune Rejection

Despite evidence for the therapeutic potential of MSCs, the mechanisms underlying the improvement in kidney function and structure remain unclear. In the past, studies have reported that injected exogenous MSCs can home into injured tubules. Consequently, it has been proposed that the ability of MSCs to transdifferentiate explains their protective effects [25]. However, if the cells act by engrafting the tubules, then either they will need to be autollogous (host-derived to prevent rejection) or the recipient will need proper immunosuppressive therapy.

Short duration of studies (usually less than a week) and rare distribution of MSCs in the kidney sections (very small in number) suggest that the beneficial effect of MSCs cannot be attributed to their engraftment or transdifferentiation. Thus, recent studies have suggested that MSCs protect against acute tubular injury through a differentiation-independent process (i.e., paracrine or endocrine process). Consequently, it was suggested that if the cells merely transit through the kidney and act in a paracrine manner to protect or stimulate the endogenous renal cells, then they might only need to survive for a few days and immune environment may not be important [16]. However, recent studies show that the situation is not so simple as it was suggested.

First, acute rejection of injected cells in cisplatin model was not evaluated nor reported. It was only reported that cells disappeared within 24 h after injection [18, 22, 31], which could suggest acute rejection. However, sensitization reaction (mixed-lymphocyte reaction—MLR test) that could confirm or omit immune reaction was not done in any of AKI studies. Since most of the studies using a cisplatin animal model investigated effects of MSC transplantation ended within 4 days, the time period may be too short for the immune reaction evaluation. Nevertheless, until now, no MLR test or immune reaction in long-term studies on AKI was reported. However, we observed the immune reaction in immunocompetent mice 3 months after MSC treatment, although mice were immunosuppressed with polyclonal antithymocyte globuline (ATG) before MSC therapy (unpublished data). MSC treatment resulted in complete restitution of cisplatin-injured organs/tissue such as the thymus, spleen, and kidney, as well as white and red blood cells (Table 5).

**Figure 1:**
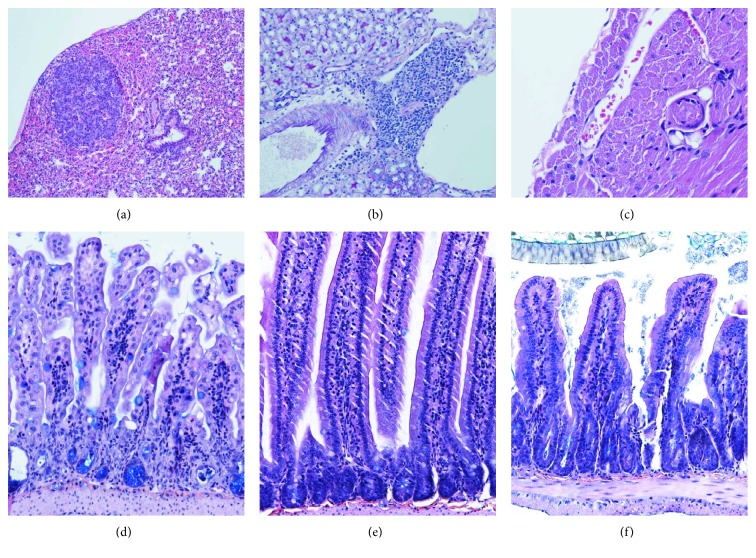
hMSC treatment in ATG immunosuppressed and cisplatin-treated BALB/cOlaHsd male mice 3 months after transplantation revealed unexpected pathology. (a) Subpleuraly, a homogeneous solid tumor (diameter 0.5 mm) with rare mitosis and uniform nuclei was sharply demarcated from the surrounding tissue in the lung of hMSC treated mouse (HE, magnification 100x). (b) Inflammatory cells (lymphocytes, plasma cells, and histiocytes) surrounding the arteriola and vein in the kidney of the hMSC-treated mouse (PAS, magnification 200). (c) Thrombus in the small artery of the right ventricle wall of ATG + cisplatin-treated mouse (HE, magnification 400x). (d) Moderate chronic jejunitis in hMSC-treated mouse—note atrophy of crypts and loss of architecture of villi (Kreyberg, magnification 400x). (e) Jejunum in the healthy untreated mouse (Kreyberg, magnification 400x). (f) Jejunum of the cisplatin-treated mouse. Restitution of the mucosa is seen; however, the height of villi is decreased compared to healthy mice (Kreyberg, magnification 400x).

However, histology revealed that the mouse had moderate chronic jejunitis (Figure 1(d)) and rare small lymphohistiocytic infiltrates in the kidneys located periglomerularly and perivascularly (Figure 1(b)) and a subpleural tumor 0.5 mm in diameter (Figure 1(a)). It is important to take into consideration that athymia is associated with profound immunodeficiency, but restitution of thymus leads to the improvement of the immune system [67]. Restitution of thymus integrity and function (which was in our case diminished following ATG and cisplatin treatment) was already described after MSC therapy [68]. Moderate chronic jejunitis and focal infiltration of mononuclear cells in lungs and kidneys found in the mouse after MSC therapy may suggest that immunoregulatory properties of transplanted MSCs together with timely vanishing effect of ATG-enabled immune system awakening and resulted in the occurrence of dispersed inflammatory changes. Thus, our case demonstrates that studying long-term MSC therapeutic effect in immunocompetent mice is challenging and may raise additional questions.

Furthermore, studies have shown that extracellular membrane vesicles (MVs) by themselves are capable of modulating T cell functions and repairing injured tissue. It was found that cytokine stimulus affects molecular mechanisms of MSCs and may have significant effects of the MV production. Kilpinen et al. [69] investigated the production of extracellular MVs from human umbilical cord blood- (UCB-) derived MSCs in the presence (MV_stim_) or absence (MV_contr_) of inflammatory stimulus (IFN-*γ*) and demonstrated that MSC paracrine regulation is complex. Although both MV_stim_ and MV_contr_ showed similar T cell modulation activity *in vitro*, only MV_contr_ were able to protect rat kidney *in vivo*. Detailed analysis of MV proteomes revealed significant differences in protein composition of MVs in dependence of the microenvironment of MSCs. MV_contr_ contained complement factors (C3, C4A, and C5) and lipid binding proteins (i.e., apolipoproteins), whereas the MV_stim_ contained tetraspanins (CD9, CD63, and CD81) and more complete proteasome complex accompanied with MHCI. IFN-*γ* stimulation of MSCs for 24 h resulted in secretion of MVs that contained the HLA-A (MHCI) molecule and both *α* and *β* units of the proteasome complex required for the antigen presentation and activation of T cells. When hUCB-MSCs were stimulated with IFNγ for 48 hours, MVs contained also HLA-II proteins. Thus, inflammatory signals in the microenvironment can significantly influence not only MSCs but also the protein content and functional properties of secreted MVs [69]. These results represent additional challenge or consideration for future studies.

## 5. What Are the Signs of Acute Cellular Rejection?

Although numerous studies have demonstrated that MSCs show low level of immunogenicity and can have an immunomodulatory role [60], animal studies have demonstrated that xenogenic or allogeneic MSCs can trigger either acute cellular or humoral immune response or both. Differentiation of MSCs (to acquire myogenic, endothelial, or smooth muscle characteristics) is associated with increased MHC-Ia and MHC-II (immunogenic) expression and reduced MCH-Ib (immunosuppressive) expression [70], which result in increased cytotoxicity in coculture with allogeneic leukocytes (acute rejection). Cells expressing MHC-Ia are usually eliminated by cytolysis, while the loss of MHC-Ib (which has been reported to suppress CD4^+^ T cell response) may result in reduced immunosuppressive effects. In animal studies, it is difficult to evaluate the signs of acute cellular rejection; thus, we stated some points that can help researchers to assess the immune reaction.

### 5.1. Beneficial or Absent Effect

The microenvironment of the damaged kidney tissue is not favorable for survival of MSCs. Cells are exposed to a hypoxic nutritionally poor environment, oxidative stress, and masses of cytotoxic factors leading to an inflammatory cytokine storm affecting the efficacy of MSC therapy. Various approaches have been investigated to help MSCs to cope/resist with the harmful microenvironment into which cells are transplanted [19, 23]. Thus, the absence of the effect of MSCs could indicate damaging microenvironment [31], MSC inactivity due to cryopreservation [71], and finally cell rejection.

On the other hand, amelioration of kidney dysfunction after MSC injection does not indicate that MSCs are not immunogenic, because along with T cell and B cell activation differentiated MSCs can stimulate the humoral immune system to produce antibodies against the allogeneic/xenogeneic cells. A good example of late rejection is the study where allogeneic or syngeneic MSCs were implanted into the infarcted rat myocardium. MSCs (versus media) significantly improved ventricular function for at least 3 months after implantation. Allogeneic MSCs differentiated by about 2 weeks after implantation, but at 5 weeks, antibodies against differentiated allogeneic MSCs (but not syngeneic) were detected in the circulation of recipient animals, and allogeneic MSCs were eliminated from the heart. Interestingly, their functional benefits were lost within 5 months [64].

### 5.2. Presence/Absence of Injected Cells—Different Method of Identification

There are various methods and markers for tracking the injected cell. They all have advantages and limitations and no single method is 100% reliable. It has been already reported that PKH26 is not a reliable tracking agent. Also, Santeramo et al. have shown that human cells (labeled with PKH26 or GFP) injected in athymic rats can give different results [18]. PKH26^+^ cells were found in the kidney close to the tubular or interstitial cells and lungs even 14 days after iv injection, while GFP^+^ cells were exclusively located in the lungs and had disappeared within 24 h after injection [18]. Obtained results confirmed that PKH26 is not a reliable tracking agent and explained the observed discrepancy among studies regarding the duration of homing of injected-labeled cells (as mentioned above in Section 3). It is important to be aware that when a labeled cell is phagocytosed by macrophages, the stain is usually not immediately degraded. Thus, the macrophage with phagocytosed fragments of labeled cells can give false positive results.

### 5.3. Presence of T Cells and/or Macrophages around Cells—Acute Rejection

Infiltration of T cells and macrophages around transplanted cells is usually a sign of acute cellular rejection. It can be observed soon after transplantation and result in the disappearance of transplanted cells within a few days. In a cisplatin animal model, immune rejection of injected cells was not studied nor reported. Therefore, conclusions about immunogenic tolerance of MSCs cannot be made. Interestingly, although the authors of one study found that injected cells became entrapped in the lungs and cells and their fragments were then phagocytosed by resident macrophages (CD68^+^) and dendritic cells within 24 h of administration, they observed the beneficial effect of injected cells on AKI [18] but did not report or mention possibility of potential acute rejection.

## 6. Risk of Tumorigenicity

Current knowledge about the risk of tumorigenicity in MSC therapy has been recently reviewed [72, 73]. It was realized that currently there is not enough data/studies to make any conclusions. “In current animal models, in which either human or animal cells (homologous models) are used, no evidence of tumor formation has been observed to date. However, the frequency of transformation of human MSCs is too low to detect overt tumor formation in established rodent model” [72]. It was also stated that “it should be emphasized that tumor formation in human patients after MSC administration has not been reported to date” [72, 73].

However, several researchers have so far described the role of MSCs in tumor formation [74, 75] and some succeeding observations of malignant lesions in the fields of transplanted MSCs [76, 77] published after the review of Barkholt et al. [72] place a serious question on the former statements.

In our case, the tumor in the lungs of the mouse was observed 3 months after MSC therapy. Cisplatin-associated lung adenomas are among already observed late onset secondary tumors in experimental rodents treated with cisplatin [56, 78]. In spite of large interspecies differences in the rates of metabolism of cisplatin, in the case of secondary solid tumors in humans (tumors that arise after treatment of primary malignancy as a consequence of cytostatic therapy), there is an average 20–40 year expected interval from the time of exposure of an individual to a chemical carcinogen until the clinical detection of a tumor [79] that raises additional questions regarding possible tumor-promoting influence of stem cell therapy.

Another important issue to consider in the context of tumorigenicity is the convenience of systemic, that is, intravenous route of MSC infusion. It was reported that only a minority of intravenously infused MSCs reaches the target tissue and then disappears after few days (see Tables 1 and 2 and Section 3 and Section 5.2) [72]. Our results showed that intravenously infused human MSCs were mostly stuck in the liver and lungs of ATG immunosuppressed mice, only few of them reached other tissues including the kidneys and intestine after MSC infusion [80]. Although MSCs mediate their effects mostly through paracrine action, massive trapping of dead and/or dying MSCs in pulmonary or liver circulation after intravenous infusion may represent some burden for ill organism. It is also not known whether all MSCs that are trapped in the lungs either die or some of them are able to survive or even transform (under special circumstances).

Probably, the most important issue associated with tumorigenicity of transplanted MSCs in this experimental model is uremic and immunocompromised status of the host. While there is not much disagreement regarding the influence of manufacturing practice and *in vitro* culture conditions, especially the duration of cell propagation on chromosomal stability of the MSCs [72] and there is also no disagreement that the immunocompromised state is predisposed to malignancies [81, 82], it is still hypothetical whether the physiological stress associated with the *in vivo* diseased environment, that is, uremic could also promote tumorigenicity in MSCs. Transplanted MSCs are believed to be confronted with cell death within a few days after transplantation due to a combination of harsh environmental conditions, anoikis, and inflammation [83, 84]. However, if not all MSCs die after transplantation but few of them were able to successfully engraft, then the survivors' exposure time to uremic environment is markedly prolonged. In the *in vitro* conditions, uremic toxins impaired human bone marrow-derived mesenchymal stem cell functionality. The harm was surprisingly not proceeded via induction of apoptosis but by promoting damage to cell membranes and altering the MSCs paracrine activity [85]. A negative influence of uremic toxins on functional characteristics of MSCs raises concern on their possible role in promoting malignant transdifferentiation of MSCs, which should be further explored.

## 7. Conclusion

Although numerous studies have shown that MSC treatment ameliorated AKI, it is important to be aware that there are many factors to consider before any conclusion about the effectiveness or safety of MSC therapy can be made. One important factor is the cisplatin model itself, because cisplatin have short-term immunosuppressive and long-term carcinogenic effects. Another important factor is stem cell quality. We have found that only few researchers used hMSC that met the proposed criteria [26]. Third factor is immune microenvironment. Many researchers used immunodeficient animals. However, not all nude mice or rats are the same or have the same immunological state. Since only few stated the exact code of animals (according to Nomenclature http://www.informatics.jax.org/nomen/strains.shtml), obtained results cannot be properly interpreted and can be misleading. Since both MSC research and cisplatin models are very complex and their underlying mechanism possess many open questions, it is of great importance to design experiments properly and state all necessary data (in accordance with ARRIVE guidelines [86] and the gold standard publication checklist [87]) to contribute to responsible conduct of animal research and to validate the results. Otherwise, it can happen that this strategy, while seems to work experimentally, will fail when applied to patients.

## Figures and Tables

**Table 1:** Xenotransplantation of human stem cells into immunocompromised or immunocompetent cisplatin-treated rodents.

Species, strain	Cisplatin treatment	Stem cell source	Stem cell treatment	Route	hMSC criteria^∗^	End	Results: effect of treatment on measured parameters	Cell tracking	Ref.
Sprague-Dawley *n* = 6	D0: 5 mg/kg, ip	hUC-derived exosomes	0.5 h prior cis 0.2 mg	rsc	No	D3	Blood: ↓ BUN, Cr, TNF*α*, IL-1*β*, IL6; kidney: ↓ histology score, TUNEL, ↑ PCNA, Bax, LC3B, BCL-2 (autophagy)	No	[7]

C57BL/6 *n* = 6-7	D0: 22 mg/kg, sc (20% mortality on D6)	hUCB versus mouse BM (ip) (third party, allogenic)	D1: 1 × 10^6^	iv/ip ip	Yes	D3	Preventive effect regardless of delivery route (xenogenic: iv/ip) or MSC source (xeno/allogeneic: ip) Blood: ↓ BUN, Cr; kidney: ↓ MCP-1, IL-6, TNF*α*, ↑ IL-10, VEGF, ≈IL-2, ↑ Treg in the kidney and spleen Mouse BM ≈ MCP-1, IL-6, IL-2, ↓ TNF*α*, ↑ IL-10, VEGF, ↑ Treg in the kidney and spleen	PKH-26: D3: observed in the kidney and spleen but not in the lung or peritoneum	[8]

C57BL/6 *n* = 6-7	D0: 20 mg/kg, sc	hUCB versus mouse BM (third party)	D3: 1 × 10^6^	iv/ip ip	Yes	D6	MSC treatment after established renal dysfunction did not show any effect	No	[8]

Sprague-Dawley *n* = 5	D0: 5 mg/kg, ip	Rat BM hAd hAF	D1: 5 × 10^6^ in 0.5 ml CM	iv	No	D4^p^ D11 D30	Comparable therapeutic effects of allogenic and xenogeneic MSCs Blood: ↓ BUN, Cr; kidney: ↓ histology score, ↓ MDA, ↑ GSH, SOD; urine: ↑ Cr clearance	No	[9]

Sprague-Dawley *n* = 15	D0: 6 mg/kg, ip	hAd	D1: 1-2 × 10^6^ in 1 ml saline	iv	No	D5	Blood: ↓ BUN, Cr; kidney: ↓ histology score, ↓ TUNEL, ↑ PCNA Urine: ↓ mALB, *β*2 mG	PKH-26 and CD105: rare around kidney tubules, frequent in the liver and spleen	[10]

C3H, female 25–30 g, *n* = 5–7	D0: 15 mg/kg, ip	hBM alone or with pFUS	D1: 1 × 10^6^	iv	No	D4	MSCs alone had weak positive effect (blood: ↓ BUN, ≈Cr; kidney: ≈histology score, ≈pAKT, ≈Ki-67, ↓ TUNEL) MSCs + pFUS had improved effect (blood: ↓ BUN, Cr; kidney: ↓ histology score, TUNEL, pAKT, Ki-67)	Human mitochondria^+^ cells: 24 h: peritubular space (4–10 MSC/field) D3: 2–4/field	[11]

C3H, female 25–30 g, *n* = 5–7	D0: 15 mg/kg, ip	hBM (pFUS)	D3: 1 × 10^6^	iv	No	D7	MSCs alone had no significant effect MSCs + pFUS had improved effect: ↑ survival; blood: ↓ dBUN, Cr	Human mitochondria^+^ cells: 24 h: peritubular space (2-fold higher number detected)	[11]

White albino rats *n* = 12	D0: 5 mg/kg, ip	hUC-derived hematopoietic stem cells (CD34^+^)	D1: 3 × 10^6^	ip	No	2 wk 4 wk	Blood: ↓ BUN, Cr, K, Na; kidney: ↑ HGF, IGF-1, VEGF, P53	PKH-26: detected in kidney Time: not stated	[12]

Sprague-Dawley *n* = 6	D0: 6 mg/kg, ip	hUCB	D1: 2 × 10^6^ in 0.5 ml saline	iv	No	D5 6 wk 8 wk	Blood: ↓ BUN, Cr; kidney: ↑ PCNA, Bcl-2, ↓ TUNEL, Bax, IL-1*β*, TNF*α*, MDA, ↓ histology score, D10: both groups returned to baseline level (BUN, Cr) 6 wk and 8 wk: structural restoration better after MSCs, increased Bax/Bcl-2 ration, ↓ TGF-*β*1, Masson, ↓ vimentin, ↑ E-cadherin	CM-Dil (cross-linkable membrane dye) *in vivo* imaging of labeled cells in kidney Time: 4 wks	[13]

C57BL/6J	D0: 10 mg/kg ip D1: 10 mg/kg ip	Mesenchymal-like progenitor cells from hESC	D3: 5 × 10^5^	iv	No	D4 **D5**^**p**^ D8 D11	Blood: ↓ BUN, Cr; kidney: ↓ histology score, TUNEL, ↑ Ki67, ↓ IL-1*β*, TNF*α*, IFN-*γ*, IL-6, TGF-*β*, MCP-1, ↑ IL-10, bFGF, IGF-1, FIGF, ≈HGF, TGF*α*, VEGF-A, VEGF-B, VEGF-C, SDF-1 D11: all groups returned to baseline level (BUN, Cr, histology)	Lipophilic carbocyanine dye DIO: 5 min, 30 min D1, D5: found in the lungs, liver, kidney D11: not found in the kidney	[14]

Sprague-Dawley *n* = 10	D0: 10 mg/kg ^M4–7^	hAd	D1: 5 × 10^5^ or D1: CM 4 ml D2: CM ml	iv ip	no	D3	Blood: ↓ BUN, Cr; kidney: ↓ histology score, TUNEL, ↓ Bax, Casp9, Casp3, p-p53, p-ERK, p-JNK, TNF*α*, COX-2, p-I*κ*B, ≈p-p38; D10: survival (20% versus 0%) CM improved BUN, Cr, histology	BrdU: D3: rarely within the tubular epithelium, also in the lung, not in the liver	[15]

BALB/c *n* = 17–60	D0: 18 mg/kg, ip	hUCB Mouse BM	D1: CM 0.5 ml once or repeated	iv	nr	D4	No effect Blood: ≈BUN, Cr, histology score, ↓ BW	No	[17]

BALB/cOlaHsd Immunosuppressed with ATG *n* = 8	D0: 17 mg/kg, ip	hUCB	D1: 5 × 10^5^ in 0.2 ml	iv	Yes	D4 D14	MSCs without ATG pretreatment had no effect MSCs with ATG pretreatment improved survival and renal functional and structural parameters Blood: ≈BUN, ↓ Cr; kidney: ↓ histology score, ≈casp-3; ↑ HO-1, GPx, ↓ SOD-1, SAA3	Dil: D2: observed mostly in the lungs and liver, rarely in the kidney and intestine	[80]

Athymic nude rats RNU (lack of T cells) Crl: NIH-Foxn1^rnu^ *n* = 6	D0: 7 mg/kg, ip	Human kidney-derived cells CD133^+^, CD24^+^, CD133^−^	D2:10^6^ in 0.5 ml PBSs (and D7: 10^6^ in 0.5 ml PBSs)	iv	No	D7	Blood: ↓ BUN, Cr, GFR; kidney: ↓ histology score (no fibrotic lesions) GFR, FITC-sinistrin (D2: increase in FITC in 62% of rats—only these rats were used for the subsequent study) D14: all groups returned to the baseline, regardless of the treatment; second injection did not improve situation; Macrophages in lungs cluster around transplanted cells, ↑ IL10	PKH26 versus GFP: D14: PKH26 cells in kidney close to tubular or interstitial cells and lungs, no evidence of GFP^+^ cells; GFP^+^ cells were located in the lungs and had disappeared by 24 h	[18]

BALB/c nude *n* = 6	D0: 10 mg/kg, D1: 10 mg/kg, ip	hAd (third party) HIF-1*α* modified	D2: 1 × 10^5^ in 0.2 ml saline	iv		D5	Blood: ↓ BUN, Cr; kidney: ↓ histology score, ↓ TUNEL, RANTES, ↓ TNF*α*, ↑ IL-10; PCR: ↑ HO-1 MSCs show protection, but HIF1ɑ-MSCs show greater impact on renal inflammatory factors and HO-1	GFP versus Cy3-labeled CK-18: rare cells overlapped showing rare differentiation of MSC into renal tubular cells CD18^+^	[19]

NOD.CB17-Prkdc scid/NcrCrl	D0: 13 mg/kg, sc	hBM reprogram into renal proximal tubular-like cells CL17	D1: 5 × 10^5^	iv	No	D4	Blood: ≈ BUN; kidney: ↓ histology score (hyaline casts and necrotic tubuli) Only slight amelioration observed. CL17 engrafted into proximal tubuli; BM MSCs found mainly at the peritubular level	hMit, cenp-a, PKH26, marker for human mitochondria and centromere protein-A; D4: tubular compartment of the kidney	[20]

NOD-SCID (Charles River) *n* = 5	D0: 13.9 mg/kg, sc	hiPSC-derived renal progenitor cells (RPC) iPSC versus RPC	D1: 5 × 10^5^	iv		D4	Blood: ↓ BUN (55%); kidney: ↓ histology score, Ki-67 RPC found in proximal tubuli and rarely in the liver, lung, and spleen, 24 h and D4 after administration iPSC failed to exert any protective effects not found in kidney or other organs 24 h after administration 8 wk: teratoma formation and analysis (*n* = 3; 10^6^/site; sc)	PKH26, human mitochondria, human nuclear antigen (HNA): 24 h and D4—kidney, liver, lung, heart, and spleen	[21]

BALB/c nude *n* = 10	D0: 14 mg/kg, sc	hUC-derived unrestricted somatic stem cells	D1: 10^5^ in 0.5 ml PBS	iv	No	D4	Blood: ↑ BUN, Cr; kidney: ↑ histology score, ~TGF-*β*1, HGF, IGF-1, ↓ VEGF-A; IFN-*γ*, and TNF*α*—not detectable in some samples D4: 3 mice died (control group), 3 mice lethargic (treated group) Worsens kidney damage	GFP^+^ cells (flow cytometry, IHC): D4: spleen, liver (not detected); lung (0.23% cells were labeled)	[22]

BALB/c nude *n* = 6	D0: 18 mg/kg, iv	hESC VEGF-modified	D1: 5 × 10^5^ in 0.5 ml	iv		D4 M2	Blood: ↓ BUN, Cr; kidney: ↓ TUNEL, histology score, ↑ PCNA, renal capillary density (CD34^+^), survival (D14; D9 critical: 60% versus 40% versus 20%) VEGF-MSCs even sign improved versus MSCs M2: No tumor detected	PKH-26: D4: small number in kidney, large number in the liver, lungs, and spleen	[23]

NOD-SCID *n* = 14–17	D0: 11 mg/kg, ip 6 h: food and water removal	hBM	D1: 5 × 10^6^ in 0.37 ml RPMI	ip		D5^p^ (D8D11D15)	Blood: ↓ BUN, Cr, ALT, amylase, phosphorous, ↓ MIP-2, G-CSF, KC, IL-1*α*, MCP-1, IFN-*γ*, GM-CSF, IL-6; kidney: ↑ Ki-67, ↓ casp3; D8: BUN returns to the baseline levels; D15: ↑survival (47% versus 10%), 2/7 mice no signal for MSCs engraftment, the same of which the BUN was not affected or not as reduced by the hMSCs	PCR specific for human 1171 bp chromosome 17-specific *α*-satellite fragment: D5: confirmed in kidney	[24]

NOD-SCID *n* = = 12	D0: 12.7 mg/kg, sc	hCB	D1: 5 × 10^6^	iv		D4	Blood: ↓ BUN; kidney: ↓ histology score, TUNEL, PCNA, peroxynitrite (oxidative stress), pAkt EM: peritubular microvascular capillary changes Survival D9–14 D40 (*n* = 3): no signs of maldifferentiation of MSCs	PKH-26: D4: peritubular areas (83%), in the context of tubular epithelium (5%) and glomeruli (12%), liver, lung, and spleen, rare or absent	[25]

^∗^hMSC minimal criteria for defining multipotent MSCs for both laboratory-based scientific investigations and for preclinical studies proposed by the International Society for Cellular Therapy [26]. N: number of animals per group; h: human; D: day; MC: minimal criteria; BM: bone marrow; Ad: adipose tissue; AF: amniotic fluid; UCB: umbilical cord blood; UC: umbilical cord; CB: cord blood; ESC: embryonic stem cells; iPSC: induced pluripotent stem cells; CM: culture media; GFP: green fluorescent protein reporter gene. ^∗^Unsorted BM MSCs: mixture of hematopoietic cells, mesenchymal stem cells, and lymphoid and myeloid progenitors. sc: subcutaneously; rsc: subcapsular; ia: intra-arterially; iv: intravenously; TUNEL: transferase-mediated dUTP nick end labeling assay; PCNA: proliferating cell nuclear antigen; pFUS: pulsed focused ultrasound; EMT: epithelial-mesenchymal transition; PKH26: lipophilic fluorescent dye; GFR: glomerulat filtration rate; FITC: fluorescein isothiocyanate (FITC)-sinistrin, (a molecule that is filtered by the kidneys, as a measure of the GFR); EM: electron microscopy; IHC: immunohistochemistry; FISH: fluorescent in situ hybridization technique of the human chromosomes; BW: body weight; mALB: microalbumin; β2 mG: macroglobulin; HIF-1*α*: hypoxia inducible factor-1*α*; p-peak (of BUN and Cr levels); ATG: polyclonal antithymocyte globuline.

**Table 2:** Transplantation of autologous, allogeneic MSCs into the cisplatin-induced animal model.

Recipient (species, strain)	Cisplatin treatment	Stem cell origin (donor)	Stem cell treatment	Route	End	Results: effect of treatment on measured parameters	Cell tracking	Ref.
*Rattus norvegicus* *n* = 12	D0: 13 mg/kg, ip	Rat BM modified MSC-hLcn2 (human lipocalin-2) (MSCs, MSC-v, MSCs-hLcn2) MSC-v: v-vector	D2: 1.5 × 10^6^ in 0.3 ml PBS	iv	D6 D23	Effect of modified MSCs on microenvironment, which is not favorable for survival of MSCs D6: no effect of any MSCs group on BUN, Cr D23: MSCs and MSC-v improved parameters, MSC-hLcn2 even more (blood: ↓ BUN, Cr, KIM-1, cystatin C, *α*GST, GSTYb1, RPA-1, histology score, ↑ AQP-1, CK-18, ↑ HGF, IGF, FGF, VEGF-1)	No	[27]

Sprague-Dawley *n* = 6	D0: 6 mg/kg, ip	Rat BM (allogeneic)	D1:	iv	D0-D5	Blood: ↓ BUN, Cr; kidney: histology score, ↓ TUNEL, Bax, ↑ PCNA, Bcl-2, ↓ miRNA-146b Study of underlying molecular mechanisms: miRNA-146b increased in AKI	Live imaging CM-Dil: Labeled cells could localize at the injury site	[28]

Sprague-Dawley *n* = 20	D0: 5 mg/kg, ip	Eat BM (allogeneic)	D1: 5 × 10^6^ in 0.5 ml CM	iv/rsc/ia	D4 D7 D11 D30	The route of administration of MSCs have no significant influence on the outcome of AKI Blood: ↓ BUN, Cr, albumin, ↑ calcium; urine: Cr clearance; Kidney: histology improved, ↓ MDA, ↑ SOD, GSH	BrdU (only rsc route): D11: in the kidney under the capsule, in the interstitium and tubules	[29]

C57BL/6J *n* = 10	D0: 12 mg/kg, ip	Mouse Ad (Syngen) Control CM Preconditioned CM	D1: 0.1 ml CM	iv	D3	Effects of CM from AdMSC preincubated in a hypoxic environment (preconditioning) Blood: ↓ N-GAL, Cr, proteins: ↓ IL-1*β*, IL-6, ≈TNF*α*; Kidney: ↓KIM-1, HMGB-1; ≈survival	No	[30]

C57BL/6J C57BL/6-Tg(CAG-EGFP)C14-Y01-M131Osb)-GFP *n* = 25	D0: 17.5 mg/kg, ip	Mouse BM (Syngen) Unsorted^∗^	D3: 1 × 10^6^ in 0.2 ml sterile PBS	ro	D10	≈Survival; blood: ≈Cr, BUN; kidney: ≈interstitial fibrosis (Masson), PAS, HE, sirius red, apoptosis, proliferation (almost absent), IGF-1 No effect during the acute phase	GFP, CFSE (flow cytometry, qRT-PCR): No labeled cells observed	[31]

Wistar	D0: 5 mg/kg, ip	Rat BM (allogenic) Nrf2-MSCs, aden-MSCs (aden-adenoviral mediated)	D1: 2 × 10^6^	iv	D3 D6 D8 D11	Modified MSC-Nrf2 (overexpressed Nrf2-nuclear factor erythroid-2 related factor 2) D6-D8: Blood: ↓ BUN, Cr; kidney: histology preventive effects	No	[32]

Sprague-Dawley *n* = 18	D0: 7 mg/kg, ip	Rat fetal kidney SC (allogenic)	D5: 2 × 10^6^ in 0.15 ml saline	iv	D8 D12	Blood: ↓ BUN, Cr; kidney: ↓ histology score, ↓ TUNEL, ↑ PCNA, ↑ Capillary density, protein ↑ HIF-1*α*, VEGF, eNOS	PKH26: D7: renal tubules and capillaries	[33]

Wistar *n* = 10	D0: 6 mg/kg, ip	Rat BM (allogenic)	D1: 2 × 10^6^	iv	D7	Comparison of BM MSCs and angiotensin II receptor blocker Blood: ↓ BUN, Cr; kidney: ↓ proteins TNF*α*, MCP-1, expression ↓ NF*κ*B, p38-MAPK, casp3, Bax	No	[34]

Sprague-Dawley *n* = 8	D0: 6 mg/kg, ip	Rat BM (allogenic)	D1: 1 × 10^6^ in 0.5 ml saline	iv	D4	Therapeutic antiapoptotic mechanisms of action of BM Blood: ↓ BUN, Cr; urine: ↓ microalbumin, ↑ Cr; Kidney: ↑ PCNA, ↓ TUNEL, histology score	PKH26: D4: peritubular areas, rarely within the tubular epithelium	[35]

C57BL/6J C57BL/6-TgCAG-ECFP/1Osb/J GFP expressing	D1: 12 mg/kg, sc	Mouse BM (syngenic)	D0: (3 different routes) 5 × 10^5^, iv 4 × 10^6^, ip (microcarriers 1 × 10^6^, sc (laparotomy)	iv/ip/ rsc	D3 D5 D8	Evaluation of organ biodistribution of transplanted MSCs Blood: ↓ BUN; kidney: histology score independent of the route of MSC delivery Distribution: 1 h after iv: trapped in the lungs (67.2%), liver (12.5%), spleen (11.4%), and kidney (5.4%); survived longer in renal subcapsular space and peritoneal cavity	Radiolabeled ^111^Indium-oxine MSCs, GFP: iv delivery: detected 24 h but not 7 days after transplantation	[36]

F344 *n* = 5–12 Heminephrectomy	D0: 7 mg/kg, sc	Rat stromal vascular fraction (SVF) from subcutaneous adipose tissue (autologous)	D1: 1 × 10^6^	rsc/ ip	D6 D14	SVF can be obtained readily without culturing and may be clinically applicable Blood: ↓ Cr (D4-D8 peak than the levels return to the baseline); kidney: ↓ TUNEL (medulla only), ↑ VEGF (cortex only), ↑ HGF, ↑ renal capillary velocity (D14), ↑ Ki67 ip administration: no effect VEGF staining localized mainly around the CFDA^+^ cells	CFDA: D14 (only rsc injection): found in the subcapsules, not located in the tubular cell layer nor in the vascular cell layer	[37]

BALB/c (female) *n* = 9–30	D0: 14.7 mg/kg, sc	Mouse BM Epo gene-enhanced (allogeneic: male C57BL/6: MHCI^+^, MHC-II^−^) MSCs/Epo-MSCs	D1: 5 × 10^6^ in 0.37 ml RPMI	ip	D4^p^ D14	Investigate the beneficial effects of Epo-secreting MSCs D4: blood: ↓ BUN, ALT in both, ↓ Cr, amylase only in Epo-MSCs; kidney: ↓ Casp3, ↑ Ki-67 in both D8-14: ↑ survival (67%/44% versus 33%), ↓ BUN only in Epo-MSCs; protective effects in liver, pancreas as well	Y chromosome-specific fragment of 444 bp (PCR of kidney): detected	[38]

HO-1^+/+^; HO-1^−/−^ (C57BL/6xFVB)F_n_ *n* = 10–13	D0: 20 mg/kg, ip	Mouse BM (syngeniec) CM of HO-1^+/+^, HO-1^−/−^	6 h after cisplatin 0.2 ml of CM concentrated 10x	ip	D3	CM of HO-1^−/−^ no effects CM of HO-1^+/+^: ↓ BW loss, blood: ↓ Cr; Kidney: ↓ histology score, casp3	No	[39]

C57Bl/6 *n* = 3	D0: 10 mg/kg, D1: 10 mg/kg, ip	Mouse BM, mouse Ad (syngeniec)	D2: 2 × 10^5^ (BM) 1 × 10^5^ (Ad)	iv/ip	D3 D6	↑ survival; blood: ↓ BUN, Cr; kidney: ↑ PCNA, BrdU, ↓ TUNEL Reduced severity of AKI seen in both BM and Ad and regardless of delivery route (iv/ip).	Y chromosome (fluorescence *in situ* hybridization) 1 h: detected in the lung, but not in the liver, spleen, or kidney 24 h and 96 h: not found in the kidney	[16]

Dog, beagles	D0: 5 mg/kg, iv	Dog BM (autologous)	D0: (after cisplatin) 1 × 10^6^ in 10 ml saline, cephalic vein	iv	D0-D4	Biochemical analyses, urinalyses, blood picture, Blood: leukopenia, ↑ BUN, Cr, phosphate, Kidney: ↑ PCNA, ↓ TNF*α*, TGF-*β*, ≈TUNEL, ≈VEGF, histology score, SD-1, HGF No improvement in the renal function, less fibrotic change in the kidney	SPIO labeled-BM: D4: not seen under MRI but detected by Prussian blue staining of kidney sections (only glomeruli but not renal tubules)	[40]

Rhesus *Macaca mulatta* monkey	D0: 5 mg/kg, iv	Monkey BM (autologous)	D4: 5 × 10^6^ or M6: 5 × 10^6^	ia	M1, M3, M6 (M9)	Preventive versus stable model (application of MSCs 6 months after cisplatin) Preventive: blood: ≈BUN, Cr, K, Na; kidney: ↓ histology score; stable: ≈ BUN, Cr, K, Na; kidney: ≈histology score No adverse effects on the spleen, lungs, and liver observed; hepatic sinusoidal dilatation and congestion in the control group after 9 months (1/15)	No	[41]

Monkey *Macaque mulatta*	D0: 5 mg/kg, iv	Monkey BM (autologous)	D4: 5 × 10^6^ cells/kg BW	ia	D4 D10 D14 D21 D28 D84	Blood: ↓ BUN, Cr, urine: ↑ Cr, urea clearance, ≈Na, K, no protenuria, polyuria up to D84; ↑ Foxp3^+^ T regulatory (Treg) cells at D28 (transplantation group only), interstitial fibrosis and matrix (Schiff staining) Biochemical improvement but no significant histological improvement	SPIO labeled-BM: seen under MRI (2 h, 24 h) and detected by Prussian blue staining (D1, D2, D28) of kidney sections (mostly localized in the glomeruli)	[42]

N: number of animals per group; D: day; BM: bone marrow; Ad: adipose tissue; CM: culture media; GFP: green fluorescent protein reporter gene. ^∗^Unsorted BM MSC: mixture of hematopoietic cells, mesenchymal stem cells, lymphoid and myeloid progenitors. sc: subcutaneously; rsc: subcapsular; ia: intra-arterial; iv: intravenously; ro: retro-orbital injection; M: month; TUNEL: transferase-mediated dUTP nick end labeling assay; PCNA: proliferating cell nuclear antigen; PKH26: lipophilic fluorescent dye; GFR: glomerulat filtration rate; IHC: immunohistochemistry; BW: body weight; CFDA SE: carboxyfluorescein diacetate, succinimidyl ester; SPIO: superparamagnetic iron oxide; ^p^-peak (of BUN and Cr levels).

**Table 3:** The acute lethal single dose of cisplatin varies among various strains of mice and rats.

Strain (origin), sex, age	Cisplatin dose	Mortality	Time	Ref.
Wistar rats female	10.8 mg/kg (9.1–12.8 mg/kg)	50%	D10	[43]
Fischer 344 rats female, 8 wks	11 mg/kg	50%	D6	[44, 45]
BALB/c (Harlan) female	14.5 mg/kg	100%	D7	[46, 47]
C57BL/6 (Japan) Male, 11–15 wks	15 mg/kg	100%	D10	[48]
Swiss Webster male	16.0 ± 0.8 19.5. ± 0.8	50% 100%	D10	[49]
C57BL/6 x DBA/2 (F1) male	10 mg/kg 14 mg/kg	0% 90%	D8	[49]
DBA2 mice female	10.7 mg/kg 16 mg/kg	50% 90%	D10 D30	[43]
129SV Male, 8–12 wks	14 mg/kg	70%	D7	[50]
CBA (UK) Male, 6 wks	10 mg/kg 15 mg/kg	0% 67%	D15	[51]
CBA mice Female, 4–8 wks	16 mg/kg	40%	D8	[52]
CBA mice Female, 24 months	16 mg/kg	100%	D7	[53]
NMRI mice female	17.0 mg/kg (14.9–19.7 mg/kg)	50%	D10	[43]

D: days after cisplatin injection.

**Table 4:** Examples of immune reaction after xenogenic or allogeneic MSC transplantation.

MSC origin	Recipient, route of transplantation	Adverse immune reaction	Ref.
Allogeneic Ad or BM	Healthy horses; intravenous injection, 3 times D0, D14, D28	Day 35: ↑ circulating CD8^+^ T cells after multiple iv injections of BM MSCs	[6]

Xenogeneic hBM	Sprague-Dawley rats: Intracardiac injection (i) Immunocompetent (ii) Immunosuppressed (tacrolimus) (iii) RNU athymic rats	↑ macrophages in myocardium of immunocompetent rats from day 2 to day 7; MLR test (peripheral blood of rats mixed with 1% or 10% of MSCs) showed ↑ lymphocyte proliferation in SD rats previously exposed to MSCs	[62]

Xenogeneic hESCs	Healthy mice, immunocompetent	Infiltrates of T cells and macrophages around injected MSCs; MSCs disappeared 3 days after transplantation (acute rejection)	[58]

Xenogeneic hBM	Rats Intracardiac injection RNU athymic rats RNU + tacrolimus Fisher + tacrolimus	Cells were present 6 weeks after transplantation in RNU rats with additional immunosuppression, in RNU rats without additional immunosuppression (tacrolimus) or in Fisher rats with immunosuppression no surviving hMSCs were found	[63]

Allogeneic or syngeneic BM MSCs	Wistar and Lewis rats; Intracardiac injection immunocompetent	Allogeneic MSCs caused T cell and B cell activation and stimulated the humoral immune system to produce antibodies against the allogeneic cells—function was lost after 5 months	[64]

Allogeneic, syngeneic, and third party BM MSCs	BALB/c or B6 mice; Sublethally irradiated mice intravenous injection	The addition of host (syngeneic) MSCs enhanced engraftment, while the infusion of donor (allogeneic) MSCs was associated with increased rejection of allogeneic donor BM cells and induce a memory T cell response. Third-party MSCs had a neutral effect on engraftment.	[65]

Allogenic donor/recipient MSCs	Rats: Lewis (donor), ACI (recipients); heart transplantation with or without immunosuppression (CsA)	Allogeneic MSCs did not prolong allograft survival. Treatment with low-dose CsA and MSCs accelerate allograft rejection in a rat heart transplant model	[66]

BM: bone marrow; Ad: adipose tissue; hESC: human embryonic stem cells; CsA: cyclosporine A; Third party: commercially available; MLR: mixed-lymphocyte reaction; RNU: Rowett nude rats (athymic with the genotype rnu/rnu).

**Table 5:** Body weight, relative weight of organs, and blood parameters in BALB/cOlaHsd mice 3 months after hMSC transplantation.

Parameter	CIS	hMSCs	Healthy
WBC (10^3^/mm^3^)	8.1	10.9	10.1
RBC (10^6^/mm^3^)	7.46	10.35	9.46
PLT (10^3^/mm^3^)	1303	773	789
Body weight (g)	23.7	31.4	31.6
RW of the spleen	0.877	0.42	0.35
RW of the liver	6.9	5.0	4.7
RW of the kidney	1.5	1.36	1.82
RW of the lungs	1.14	1.14	1.25
RW of the heart	0.96	0.67	0.59

CIS: mice treated with ATG and cisplatin (ip, 17 mg/kg); hMSCs: mice treated with ATG, cisplatin (ip, 17 mg/kg), and hMSCs (iv, 0.5 × 10^5^ cells in 0.2 ml PBS); healthy: mice received PBS instead; WBC: white blood cells; RBC: red blood cells; PLT: platelets; RW: relative weight (weight of organ divided by body weight ^∗^100).
